# Sensitive and effective electrochemical determination of butenafine in the presence of itraconazole using titanium nanoparticles-ionic liquid based nanocomposite sensor

**DOI:** 10.1007/s11696-022-02593-3

**Published:** 2022-12-05

**Authors:** Mona A. Mohamed, Nahla N. Salama, Maha A. Sultan, Hadeer F. Manie, Maha M. Abou El-Alamin

**Affiliations:** 1 Pharmaceutical Chemistry Department, Egyptian Drug Authority (EDA), Cairo, Egypt; 2 Department of Pharmaceutical Analytical Chemistry, Faculty of Pharmacy, Helwan University, Cairo, Egypt

**Keywords:** Butenafine determination, Modified electrochemical sensors, Titanium nanoparticles, Ionic liquid

## Abstract

The evaluation of the bioavailability of topically applied medications that act inside or under the skin is a challenging task. Herein, the current study describes a simple, quick, and low-cost electrochemical platform for determining butenafine hydrochloride (BTH) that is mainly prescribed as a treatment of dermatophytosis, applying titanium nanoparticles and an ionic liquid as outstanding mediators. In terms of low detection limits (61.63 nM) and extensive range of 2.21 × 10^–7^–13.46 × 10^–5^ M, the established electrochemical technique provided worthy analytical performance for butenafine hydrochloride sensing. The suggested sensor's practical applicability was effectively demonstrated in pharmaceutical preparations, actual stratum corneum samples, and simultaneous detection of butenafine hydrochloride and Itraconazole in pharmaceutical preparation for the first time. All of the experimental factors, like the pH and scan rate, have been investigated and optimized. Diffusion coefficients of butenafine hydrochloride at bare and modified sensors were calculated.

**Supplementary Information:**

The online version contains supplementary material available at 10.1007/s11696-022-02593-3.

## Introduction

Butenafine hydrochloride (BTH) is a benzylamine antifungal drug with great efficacy of treatment and a long duration of action when administered topically against various mycoses (Chiou et al. 2021). The severe acute respiratory syndrome coronavirus 2 (SARS-CoV-2) proteolytic activity has been discovered to be blocked by BTH (Chiou et al. 2021). At doses less than 10 μM, butenafine inhibited SARS-CoV-2 activity by 50% (Chiou et al. 2021). BTH has the chemical name N-(4-tert-butylbenzyl)-N-methyl-naphthalenemethylamine hydrochloride (Nahm et al. 1999). BTH, similar to the allylamine medications, inhibits squalene epoxidase (a sterol synthesis enzyme, which transforms squalene to ergosterol), causing squalene to accumulate intracellularly. Fungal cell membranes are disrupted because of this accumulation. Besides, BTH possesses an anti-inflammatory property in vivo. After UVB treatment, it minimizes cutaneous erythema (Nahm et al. 1999).

In the soil and other parts of the environment, there are many distinct forms of fungal germs. They can act directly on the surface of the body, infecting the lips, nails, and skin, as well as causing a fungal infection. The use of nanocarriers as a topical therapy for fungal infections has shown to be a feasible delivery strategy (Mahdi et al. 2021).

BTH demonstrates a high level of fungicidal activity, particularly against dermatophytes, aspergilli, dimorphic, and dematiaceous fungi. Following topical administration, the medication exhibits great penetration into the epidermis and a lengthy retention duration, imparting residual therapeutic efficacy after treatment discontinuation. For tinea pedis, tinea corporis, and tinea cruris, topical butenafine 1% cream has been shown to be effective for short-term treatment (Sakagami 2010).

To best of our knowledge, different analytical techniques were used to determine BTH including HPLC (Ankam et al. 2009; Bhosale and Rajput 2011; Miron et al. 2014), LC (Barth et al. 2011a, 2011b; Song et al. 2011), and potentiometric methods (Ali et al. 2020; Frag etal. 2020).

Itraconazole (ITZ) is an antifungal medication taken orally that inhibits cytochrome P450-dependent enzymes, causing ergosterol production in fungal cell membranes to be inhibited (Feng 2003). ITZ is used in combination with BTH cream to treat pityriasis versicolor and reduce the long-term recurrence rate without causing any noticeable side effects (Hong-Xiao et al. 2010). Simultaneous determination of ITZ and BTH is therefore regarded as a new and promising field of research.

Compared to other techniques, electrochemical methods are advantageous over other options since they allow for faster, cheaper, and cleaner analysis as well as high sensitivity (Mohamed et al. 2021, 2020). Analytical methods that use chemically modified electrodes to detect minute concentrations of physiologically relevant substances have long been considered very sensitive and specific (Mohamed 2018; Mohamed et al. 2016; Saadeh et al. 2018; Ghalwa et al. 2020).

In recent years, nanoparticles have gained a lot of attention because of their use in biosensing technology for the detection of different pharmaceuticals. Electrochemical sensing processes have been shown to be useful in the detection of toxins, environmental management, and food safety (Mohamed 2018; Ghoniem et al. 2021).

Because of its high aspect ratio, optical transparency, exceptional biocompatibility, and relatively robust conductivity, titanium dioxide nanoparticles have been frequently employed in voltammetry for the research of various analytes (Sanghavi and Srivastava 2013; Mohamed et al. 2017a).

Ionic liquids have lately attracted a lot of attention in a variety of fields, notably electrochemistry, high viscosity and nonvolatility, as well as strong ionic conductivity, thermal stability, oxidative and thermal stability, huge solvation power, and non-flammability are some of its distinctive qualities (Mohamed et al. 2019).

In the present work, we first report the determination of BTH in a drug substance and pharmaceutical preparation using Ti nanoparticles and IL. We also present some new insights into the attractive square wave voltammetry (SWV) of this electrode for the simultaneous determination of BTH and ITZ.

## Experimental

### Instrumentation

The electrochemical characteristics of the samples were analyzed using the Bio-logic SP 150 electrochemical workstation. A C3-stand from BASi research products (USA) was used to connect a one-compartment cell with a three-electrode setup to the electrochemical workstation. As an auxiliary electrode, a platinum wire, while carbon paste electrode (CPE) was used as working electrode, and Ag/AgCl (3.0 M NaCl) was used as reference electrode. All electrodes were obtained from BASi research products (USA).

For pH measurements, a Cyberscan 500 digital (EUTECH Instruments, USA) pH-meter with a glass combination electrode was used.

Electrochemical impedance spectroscopy measurements were performed over a frequency range of 100 mHz to 100 kHz. All electrochemical experiments were carried out at a temperature of 25 °C.

### Materials and reagents

Western Pharmaceutical Industries generously provided BTH (B.N. 83160221). According to the manufacturer's certificate of analysis (COA), the BTH assay was 99.50 percent accurate (HPLC). 1-Hexyl-3-methylimidazolium hexafluorophosphate (IL) was obtained from Sigma-Aldrich. Butaximark® was obtained from the market and includes 10.00 mg BTH/1.00 gm.

Aldrich provided graphite powder with a particle size of 50.0 μm. For the manufacture of the paste electrodes, paraffin oil (Merck Co. Germany) was employed as the pasting liquid. As a supporting electrolyte, 0.04 M Britton–Robinson buffer (B–R buffer) was used. To achieve the necessary pH, phosphoric, acetic, and boric acids (Sigma-Aldrich) were combined with 0.20 M NaOH (2.0–6.0). All of the solutions were made with arium® pro, sartorius (Goettingen, Germany) ultra-pure water.

### Preparation of different electrodes

Using a pestle and mortar, graphite powder (0.50 g) and paraffin oil (0.30 mL) were mixed to make a carbon paste electrode (CPE). To get a shining look, the carbon paste was packed into the hole of an electrode body, scraped out the excess with a typical paper, and the electrode was polished on a smooth paper.

A mixture of 985.00 mg graphite powder, 15.0 mg Titanium nanoparticles (Ti), and the 5 μL ionic liquid was used to make the improved sensor.

Using a mortar, the mixture was gently homogenized for 45.0 min. The paste was then made by adding 0.60 mL of paraffin oil. The paste was loaded into the electrode's hole and smoothed out with filter paper until it was gleaming. By removing the surplus paste from the syringe and republishing it with weighing paper, a new surface was obtained.

Ti-IL/CPE was the name given to the modified electrode. Various modified carbon pastes containing various percentages of Ti nanoparticles (0.5, 1.0, 1.5, and 2.0 percent w/w) were made in the same way.

### Standard and working solutions

A stock solution of BTH (1.0 × 10^–2^ M) was prepared by dissolving 353.93 mg in ethanol HPLC-grade (Sigma-Aldrich) in a 100.0-mL volumetric flask.

Working solutions for BTH were made by diluting the stock standard solution with ethanol to obtain solutions in the concentration range of 1.0 × 10^−3^ to 1.0 × 10^−4^ M. At 4 °C, these solutions remained stable for roughly a week.

### Experimental procedure

The modified electrode Ti-IL/CPE was cycled between 0.0 and 1400.0 mV with a scan rate of 100.0 mV s^−1^ in 0.04 M B–R buffer solution of pH 2.0 numerous times before any voltammetric measurements were taken until a repeatable response was attained. The modified Ti-IL/CPE electrode was then placed in a new cell containing 0.04 M B–R buffer at pH 2.0 and the appropriate concentrations of BTH. With a scan rate of 100.0 mV s^−1^, cyclic voltammograms (CV) were recorded between 0.0 and 1400.0 mV.

### The recommended procedure for calibration curves

Using a micropipette, aliquots of BTH ranging from 2.21 × 10^–7^ to 13.46 × 10^–5^ M were put into a series of 5-mL volumetric flasks, and the volume was filled to the mark with 0.04 M B–R buffer pH 2.0. After transferring the solution to the electrolytic cell, a square wave voltammogram was obtained. The SWV analytical process for determining BTH was verified according to the International Conference on Harmonization (ICH) principles (Zhang et al. 2020).

### Application to pharmaceutical formulations

A sufficient amount of Butaximark® cream equivalent to a stock solution of concentration 1.0 × 10^−3^ M of BTH was weighed, transported to a 100-mL calibrated flask, and dissolved in 50 mL of ethanol.

The solution was sonicated at 40 °C for 45 min, then allowed to cool to ambient temperature before being filtered through 0.45 μm filter paper. 20 µL was accurately transferred to a 5.0-mL volumetric flask, which was then filled to the mark with 0.04 M B–R buffer pH 2.0.

The concentrations of the cited medication were estimated using the recommended approach listed under "Recommended procedure for calibration curves." The accuracy of the proposed method was assured by the application of the standard addition technique.

### Application to real samples

#### Tape stripping

One healthy female volunteer (30 years of age), with no history of dermatological diseases, was chosen for this study. She was informed not to apply any cosmetic treatment to the test area on her forearm 24 h prior to the start of the study. 30 min before the experiment, the volar aspect of the forearm of each subject was cleaned. Using adhesive labels, the application site was marked on the forearm keeping one site as a blank (dimensions of each area 2 × 5 cm^2^) (Abdel-Salam et al. 2017).

Approximately, 5 mg/cm^2^ of 1% Butaximark® cream was applied uniformly on the selected site by means of a glass rod (Herkenne et al. 2008). After 12 h of application, the residual formulation was removed by gentle wiping with cotton swab (Parfitt et al. 2011).

**Scheme 1:**
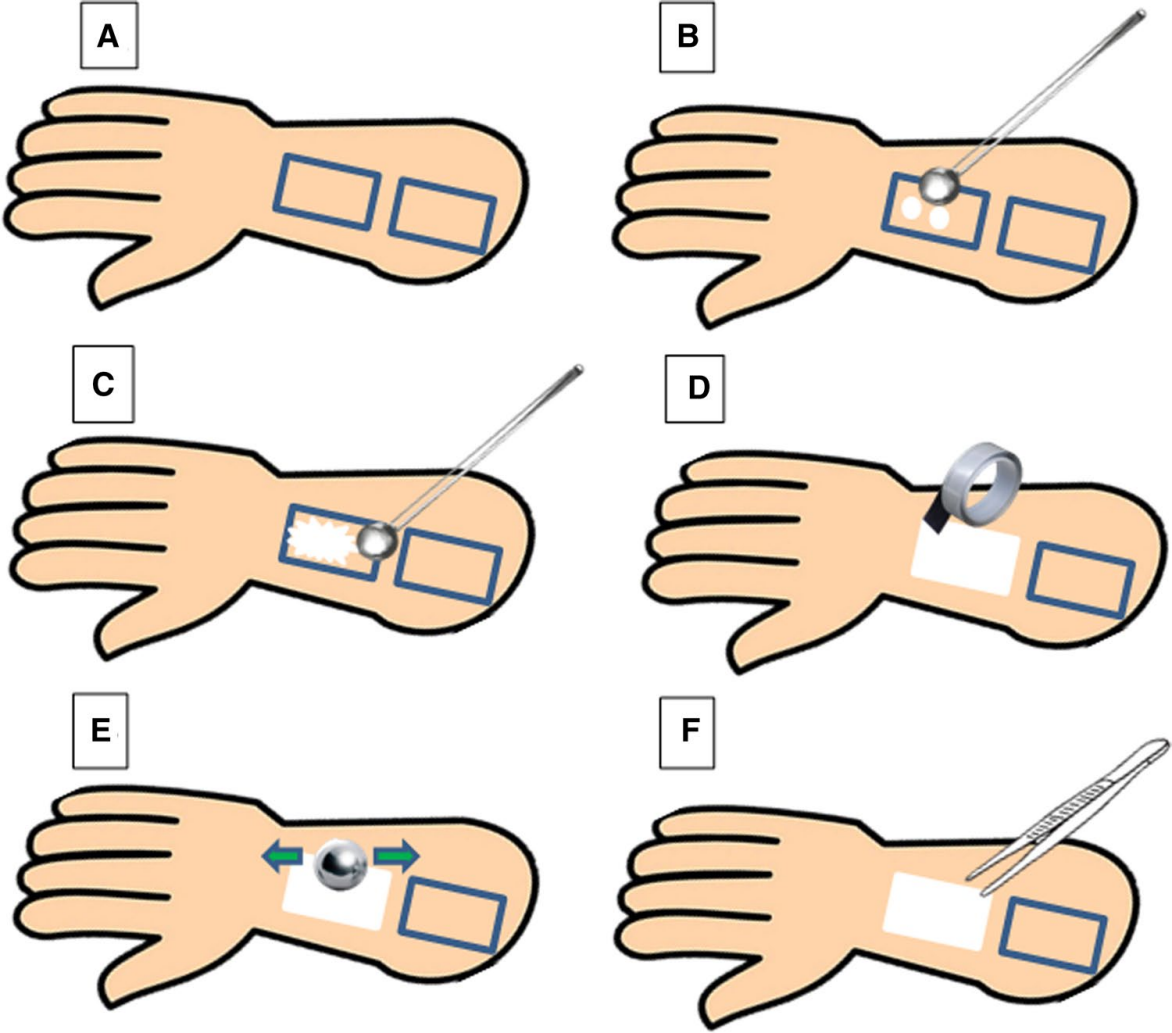
Steps of the tape stripping method: **A** marking the sites using adhesive labels, **B** application of the cream on the marked skin areas, **C** homogeneous distribution of the cream, **D** adhesive tapes are applied after each duration, **E** adhesive tape are pressed with a roller on the skin and **F** stripping of the tape

The TS procedure was performed utilizing 15 adhesive tape strip at each site (Au et al. 2010) using Scotch TM tape (No. 845 Book Tape 3 M company, St. Paul, MN). The tape was pressed with a 100 g roller applied back and forth for 10 times to remove any furrows and wrinkles on the TS procedures (Teichmann et al. 2007). The tapes were removed with a rapid movement, changing the direction of the removal of each strip in order of clockwise rotation. Scheme 1 displays the steps of the tape-stripping method.

### Collecting samples

The 15 strips were combined and extracted over 16 h in 10 ml ethanol. The obtained extract was filtered through 0.2 μm pore size syringe filter. 0.5 ml of the filtrate was accurately transferred to a 5.0-mL volumetric flask and the volume was completed to the mark with 0.04 M B–R buffer pH 2.0. The recommended procedure mentioned under," Recommended procedure for calibration curves" was followed and the concentrations of the cited drug were calculated. The accuracy of the proposed method was assured by the application of standard addition technique.

## Results and discussion

### Characterization using SEM

In order to characterize the surface morphology of the electrodes, scanning electron microscopy (SEM) is considered a critical approach to getting vital information about the electrode surface morphology (Mohd Norsham et al. 2020).

**Fig. 1:**
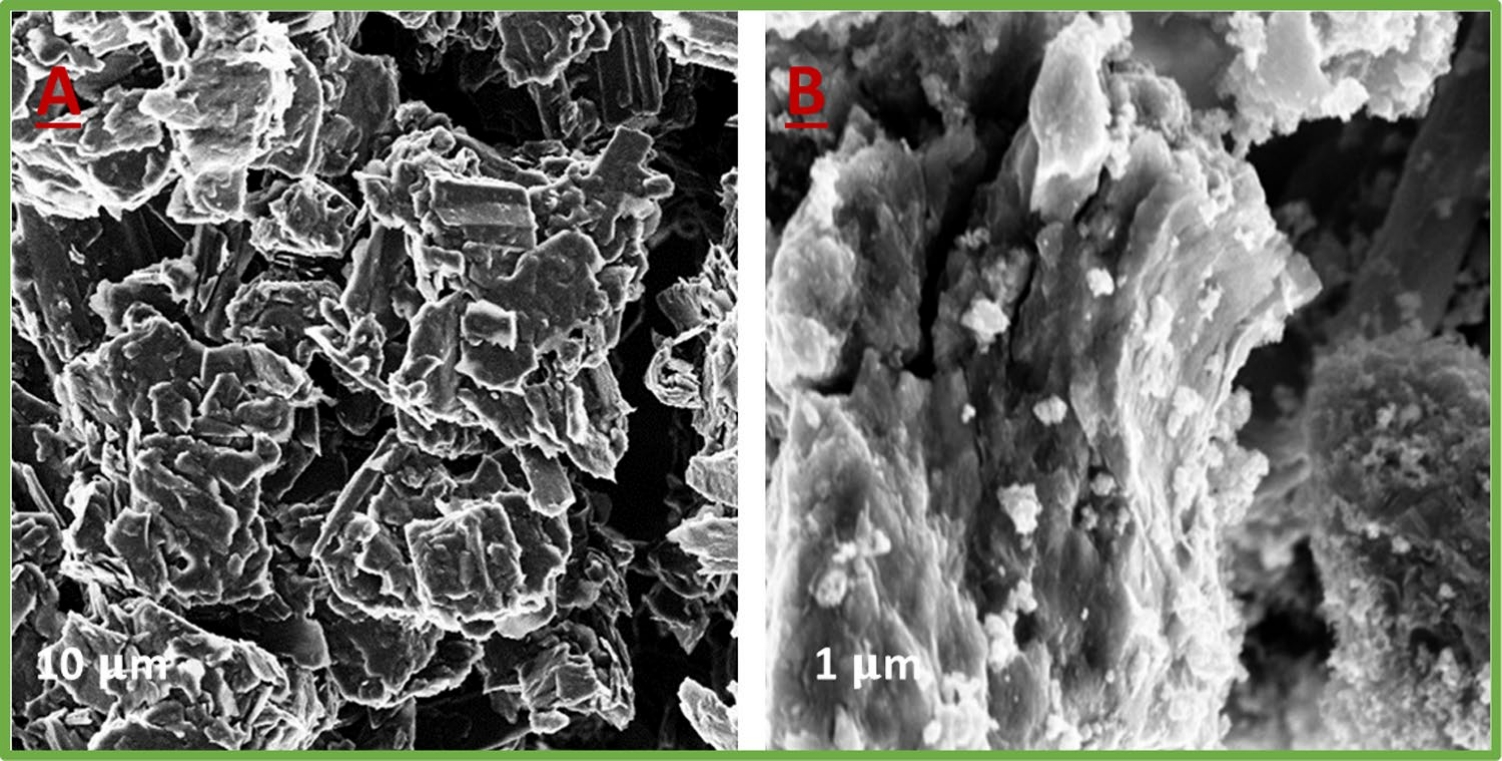
SEM images of the **A** Ti/CPE and **B** Ti-IL/CPE

Figure 1 shows how scanning electron microscopy (SEM) data were applied to compare and define the morphological properties of various electrodes. Large graphite flakes with disorganized distribution and irregular form dominated the bare Ti/CPE surface. The gaps between the isolated graphite and Ti particles were relatively big, affecting the charges transmitted in the vertical direction of planes. While the separate carbon layers vanished from the surface of the Ti-IL/CPE, a homogeneous surface with many consistently micrometer-sized particles developed.

**Fig. 2:**
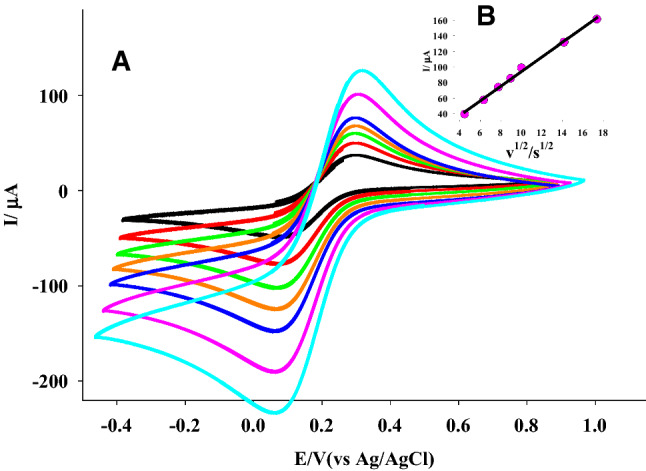
**A** CVs of Ti-IL/CPE in 5.0 mM K_3_Fe(CN)_6_ solution in 0.10 M KCl at different scan rates. **B** The anodic current─*v*^1/2^ plot at Ti-IL/CPE

Using Randles–Sevcik (Allen and Larry 2001), the surface area of the constructed sensors was measured by acquiring CVs with 5.0 mM K_3_Fe (CN)_6_ probe at various scans onto CPE and 1.50% (w/w) Ti-IL/CPE (Fig. 2). The surface areas obtained for CPE and 1.50% (w/w) Ti-IL/CPE were 0.056 and 0.257 cm^2^, respectively. The obtained facts showed that the combination of Ti nanoparticles and ionic liquid leads to increased electrode area and electrochemical activity in Ti-IL/CPE sensor (Mohamed et al. 2019, 2017b).

### Effect of Titanium nanoparticles content

The proportion of Ti nanoparticles used in sensor manufacture affects Ti-IL/CPE electrochemical behavior. As a function of Ti nanoparticles, the anodic peak current for BTH oxidation at Ti-IL/CPE electrode was investigated using the ratios from 0.5 to 2.0%. The optimal anodic peak current was obtained when the CPE was produced with 1.50% Ti nanoparticles. The background current was raised by increasing the number of Ti nanoparticles, resulting in poor reproducibility of the BTH response (Figure S1). The increase in background current is referred to the arising of non-Faradaic current. Experimental settings in fundamental voltammetric research are usually constructed so that non-faradaic contributions are quite minimal (Barrosse-Antle et al. 2010).

### Ti-IL/CPE electrochemical behavior

**Fig. 3:**
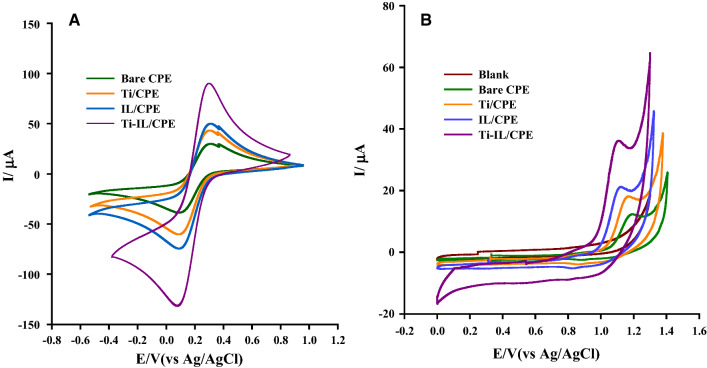
**A** CVs 5.0 mM K_3_Fe(CN)_6_ in 0.10 M KCl solution at different electrodes. **B** CVs of 0.1 mM of BTH (pH 2.0) at different electrodes

The CVs of unmodified CPE, Ti/CPE, IL/CPE and Ti-IL/CPE were examined using 5.0 mM K_3_Fe(CN)_6_ as the electrochemical probe in order to better understand the electrochemical behavior of both electrodes. Figure 3A shows that when compared to the unmodified sensor, the highest I_p_ was obtained for 1.50% (w/w) Ti-IL/CPE, which may be ascribed to its greater surface area and good electrochemical conductivity owing to the integration of Ti nanoparticles and IL. Ti-IL/CPE has 2.97 times more redox peaks than CPE.

Furthermore, CV responses in the presence of 0.10 mM BTH in 0.04 M B–R buffer were used to analyze the modified sensor's electrochemical behavior (pH 2.0). The electrochemical oxidation of BTH is represented by the anodic peak current (Fig. 3B) was 14.10 µA at 1187.0 mV when using unmodified CPE, whereas the current magnitude of BTH increased to 40.69 A at 1103.0 mV when using 1.5% Ti-IL/CPE. Comparing the electrodes responses, the Ti-IL/CPE has the highest anodic current. As a result of their strong catalytic activity, Ti nanoparticles act as a mediator, increasing the rate of electron transfer.

### pH optimization

Cyclic voltammograms were carried out on the Ti-IL/CPE using 0.1 mM BTH in a pH range of 2 to 6 of 4.0 × 10^–2^ M buffer solution.

**Fig. 4:**
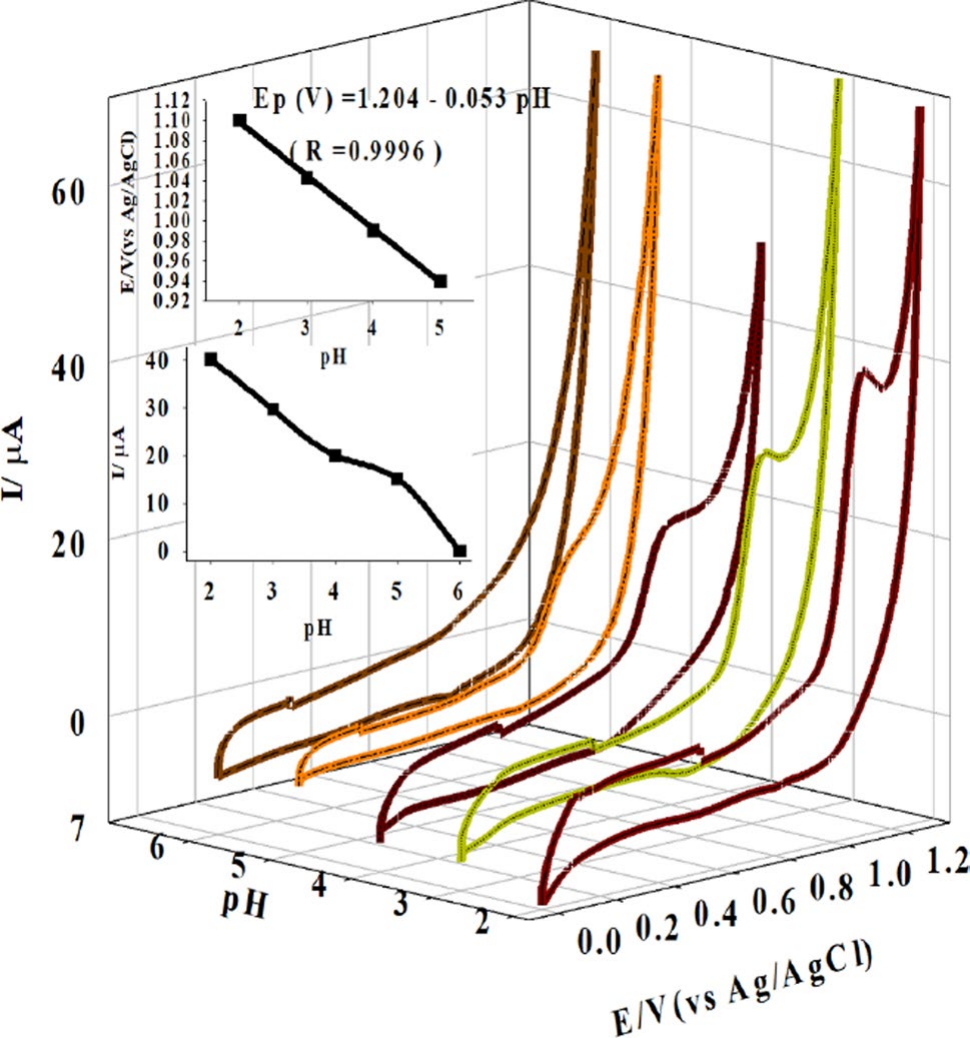
BTH (0.10 mM) voltammetric responses at different pH values using Ti-IL/CPE (*v* = 100.0 mV s^−1^). The inset displays a relation of the Ep of BTH vs. pH

The behavior of BTH at different pH levels is depicted in Fig. 4 as the peak potentials (Ep) shift to a less positive potential as the value of the pH rises and the current decreases until the peak vanishes and the clear solution becomes turbid. At pH 6.0, the peak vanishes and the clear solution becomes turbid. At pH 2.0, the greatest anodic peak current (Ip) was measured, and this pH was chosen as the best for the selective and sensitive measurement of BTH.

The voltammetric responses show a linear relationship with the buffer solution pH, which can be fit as Ep (V) = 1.204–0.053 pH; *R* = 0.9996. A well-developed anodic peak current was observed spanning the pH range of 2.0 to 5.0, as shown in (Fig. 4).

**Scheme 2:**
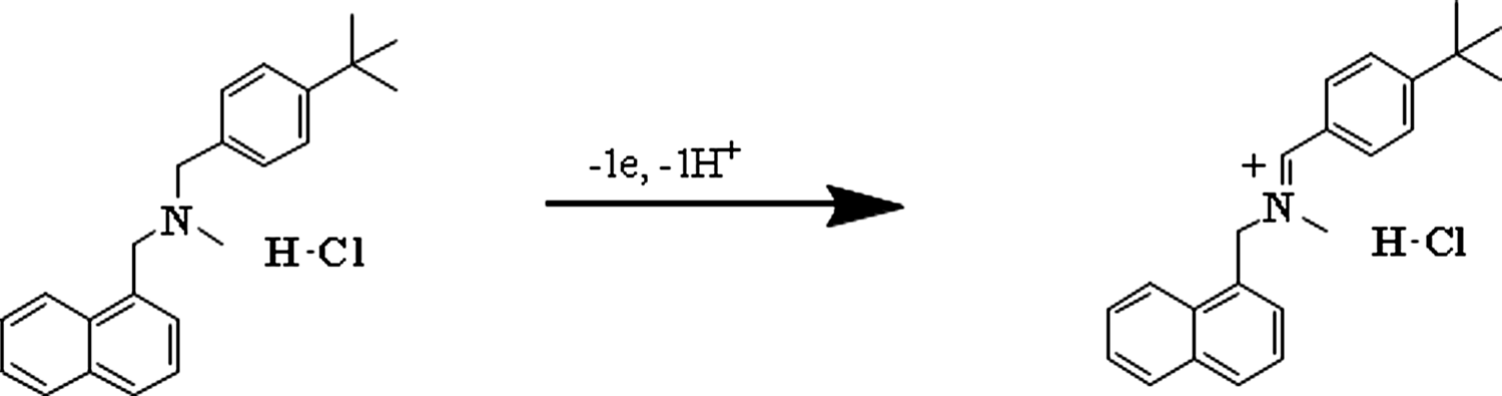
The proposed BTH oxidation mechanism

Furthermore, when the pH is raised to 5.0, the oxidation peak shifts to a lower positive potential, representing that the oxidation process is irreversible, as validated by CV analysis. According to the Nernst equation, the slope of the linear peak potential and pH correlation for BTH is 53.0 mV/pH, showing that the electrochemical oxidation process comprises the same number of electrons and protons transfer reactions. The proposed mechanism for the oxidation of BTH is revealed in Scheme 2.

### Scan rate impact

To explore the process behavior that was occurring at the sensor surface and to evaluate the electrochemical strategy of the sensor reaction, CVs of a constant concentration (0.10 mM BTH) at pH 2.0 were collected at varied scan rates (Figure S2).

The increase in scan rate was accompanied by an increase in currents (Ip). As shown in Figure S2, inset A, the Ip increased linearly with the scan rate square root, indicating that the process is diffusion regulated. For the oxidation process, the appropriate linear equation was *I*_*p*_ (µA) = 3.28 *v*^1/2^ (Vs^−1^)^1/2^ + 5.56; *R* = 0.9995. The processes prevalent at the working electrode surface are clearly diffusion-controlled, based on the foregoing findings.

As illustrated in Figure S2, inset B, direct proportionality was found between log current and log scan rate in the 20–300 mVs^−1^ range using the formula: log I (A) = 0.7422 + 0.4235 log v; *R* = 0.9994. The slope of the derived linear relationship is less than 0.5 Ti-IL/CPE, implying that the electroactive constituents are transmitted via a diffusion-controlled course, implying that the modified electrode plays a substantial role (Laviron et al. 1980).

### Chronoamperometric measurements

Cottrell's law (Yarin et al. 2014) describes the current corresponding to the electrochemical reaction (under diffusion control) for an electroactive material with the diffusion coefficient (*D*) as the following:

$$I\left( t \right) = nFAC * \left( {\sqrt {\frac{D}{\pi t}} } \right)$$

where *A* is the electrochemically active area, *D* is the diffusion coefficient, *C* * is the bulk concentration of ferrocyanide, while the rest of the parameters have their standard definitions.

The diffusion coefficient was calculated for unmodified and Ti-IL/CPE using the Cottrell equation (Yarin et al. 2014), Figure S3. The diffusion coefficient values were found to be 8.65 × 10^–6^ cm^2^ s^−1^ and 5.96 × 10^–5^ cm^2^ s^−1^for unmodified and Ti-IL sensors, respectively. The Ti-IL/CPE diffusion coefficient is greater than that of CPE, showing that the modified electrode has more diffusion capacity than the unmodified electrode. The Ti nanoparticles and ionic liquid facilitated the charge transfer and diffusion of ions through the electrode surface.

### Validation of the proposed Ti-IL/CPE

The linearity, selectivity, precision, and accuracy of the created electrochemical platform were tested in accordance with the International Conference on Harmonization (ICH) requirements (Zhang et al. 2020).

### Linearity

**Fig. 5:**
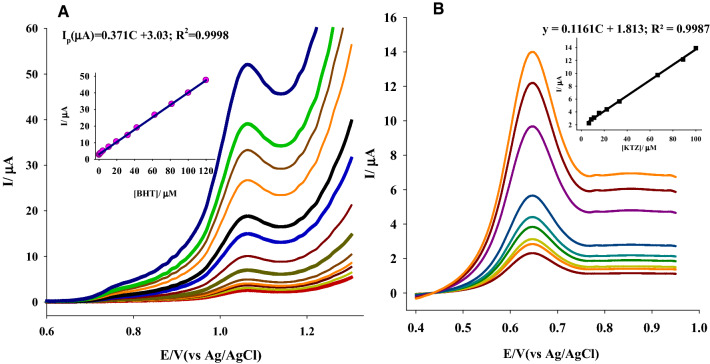
**A** The SWV using Ti-IL/CPE sensor in pH 2.0 B–R buffer corresponding to: 2.21 × 10^–7^–13.46 × 10^–5^ M BTH (*v* = 100 mV s^−1^), *P*_H_ = 0.25 V, *P*_W_ = 0.50 V, *S*_H_ = 0.02 V. B) SWV for ITZ in the concentration range 6.62 × 10^–6^–1.00 × 10^–4^ M using Ti-IL/CPE (*v* = 100 mV s^−1^), *P*_H_ = 0.25 V, *P*_W_ = 0.50 V, *S*_H_ = 0.02 V. The inserts are current vs*.* concentration plots

The BTH determination was used to validate the Ti-IL/CPE sensor that had been developed. Using the Ti-IL/CPE, it was discovered that the peak currents related to the electrochemical oxidation of BTH are linearly subordinate on BTH quantity across the range of 2.21 × 10^–7^–13.46 × 10^–5^ M, while the regression equation is found to be Ip (µA) = 0.371C + 3.03; *R*^2^ = 0.9998, Fig. 5. Table S1 shows that the suggested platform has a high level of accuracy and precision.

On the other hand, the linearity range for ITZ found to be 6.62 × 10^–6^ M to 1.00 × 10^–4^ M and the regression equation is found to be Ip (µA) = 0.1161C + 1.813; *R*^2^ = 0.9987.

### Detection and quantitation limits

The limit of detection (LOD) and the limit of quantification (LOQ) were determined to be 6.163 × 10^–8^ and 2.054 × 10^–7^ M, respectively, Table S1. By utilizing the formulas 10S/b and 3.3S/b (where *S* is defined as the standard deviation of the Ip and *b* is referring to the calibration curve slope), it is possible to calculate the calibration curve slope for both parameters.

#### Accuracy

The percent recovery of the proposed electrochemical procedure was used to assess accuracy (Zhang et al. 2020). Five repeat assays for varying concentrations of BTH in the quantification range were performed. The accuracy of the disclosed technique for the assay of BTH was demonstrated with satisfactory results. Compared to previous related methodologies, the proposed approach for evaluating the studied drug in drug substances and medical products showed statistically significant findings. In accordance with the results of Table S2, the calculated *t*- and *F*-test values were lower than the reported values, suggesting that there was no statistically significant difference in accuracy and precision.

#### Repeatability and intermediate precision

The mean RSD % (*n* = 3) was calculated after triplicate examination of three concentrations of BTH (10.98, 19.61, and 32.25 µM) on the same day (intraday test) or three successive days (interday assay). As shown in Table S3, the estimated average RSD% for the intra- and inter-day studies did not exceed 1%, demonstrating acceptable accuracy. The stability of the Ti-IL/CPE was investigated throughout an eight-day storage period in the air with no change in the voltammetric peak current. Table S3 shows that the sensor maintained 98.87–99.02 percent of its initial response after 14 days.

#### Interference studies

Because of the existence of ascorbic acid (AA) and uric acid (UA) in a variety of real samples, the electrochemical oxidation of BTH employing Ti-IL/CPE through SWVs was also examined (Ghoniem et al. 2021). Figure S4 shows how remote these are from the analytical peak of interest. Using a given drug concentration, the influence of a variety of materials that might potentially affect the electroanalytical signals of BTH was also investigated (1.0 × 10^–5^ M). Each of these drugs was added to a fixed BTH amount using the optimum pH solution, and the magnitude of the BTH signal was measured. None of the potential interfering compounds tested interfered with the electroanalytical detection of BTH. Each interfering material had a tolerance limit of less than 5.0%. Furthermore, it was shown that 100-time increased amounts of inorganic ions (e.g., sodium, potassium, calcium, zinc, magnesium, iron, Cl^−^, SO_4_^2−^, and NO_3_^−^) had no impact on oxidation peak currents.

## Analytical application of Ti-IL/CPE

### Application to the medicinal formulation

The proposed technique had been tested on a commercially available medicinal cream. Table 1 displays the results. The recommended sensor's accuracy is demonstrated by the recovery figures. According to the findings of the experiments, this sensor is extremely sensitive to identifying lower and higher BTH concentrations in the dosage form.

**Table 1:** Determination of BTH in dosage form using the proposed sensing protocol

Pharmaceutical formulations	Claimed (µM)	Recovery% ± SD (*n* = 5)	Standard addition	
			Amount added (µM)	Recovery% ± SD (*n* = 5)
Butaximark® (10 mg of BTH /1gm)	4.42		13.16	98.57
			25.97	99.86
			38.46	98.94
			66.39	99.31
Mean ± S.D		98.75 ± 0.625		99.17 ± 0.550

#### Application to real samples

As one of the advantages of topical application is that the infected area can be treated with minimum systemic toxicity but the undetectable systemic levels cause difficulty in measuring the systemic availability of the drug (Alberti et al. 2001). Therefore, to evaluate topical drug bioavailability and the effectiveness of the applied cream, the convenient metric is measuring drug concentration in the stratum corneum (SC), as antifungals targeting this organ. This is done by quantifying BTH during the sequential tape stripping process after treatment with Butaximark® cream on human subjects by SWV.

#### Tape stripping application

The concentration of BTH in the SC tissue subsequent a single topical claim of 5 mg/cm^2^ of Butaximark® to a volunteer was determined using SWVs and it was found to be around 5.26 µM/cm^2^ (average of three determinations). The results are displayed in Table 2. This relevant concentration is above the minimum inhibition concentration (MIC) for BTH, which should in clinical use result in effective inhibition of the growth of fungi (Syed and Maibach 2000; Kokjohn et al. 2003). Standard addition technique was applied to eliminate any matrix effect of the proposed method. As shown in Table 2, the mean recovery% indicates that BTH can be successfully determined in SC tissue after the topical application of the cream.

**Table 2:** Determination of BTH in the stratum corneum using the proposed sensing protocol after 12 h of Butaximark® cream application on the forearm of the volunteer

Pharmaceutical formulations	Claimed (µM/cm^2^)	Recovery% ± SD (*n* = 5)	Standard addition	
			Amount added (µM)	Recovery% ± SD (*n* = 5)
Butaximark® (10 mg of BTH /1gm)	5.26		22.31	99.48
			53.19	99.54
			72.73	101.41
			91.48	98.13
			109.48	99.72
			126.78	99.14
Mean ± S.D		98.47 ± 0.853		99.57 ± 1.06

#### Application to the simultaneous determination of butenafine hydrochloride and Itraconazole

According to the literature, no study has been published that uses electrochemical techniques to simultaneously determine BTH and Itraconazole (ITZ). The detection of BTH and ITZ in pharmacological compounds concurrently utilizing the Ti-IL/CPE technology is thus a significant element of our research.

**Fig. 6:**
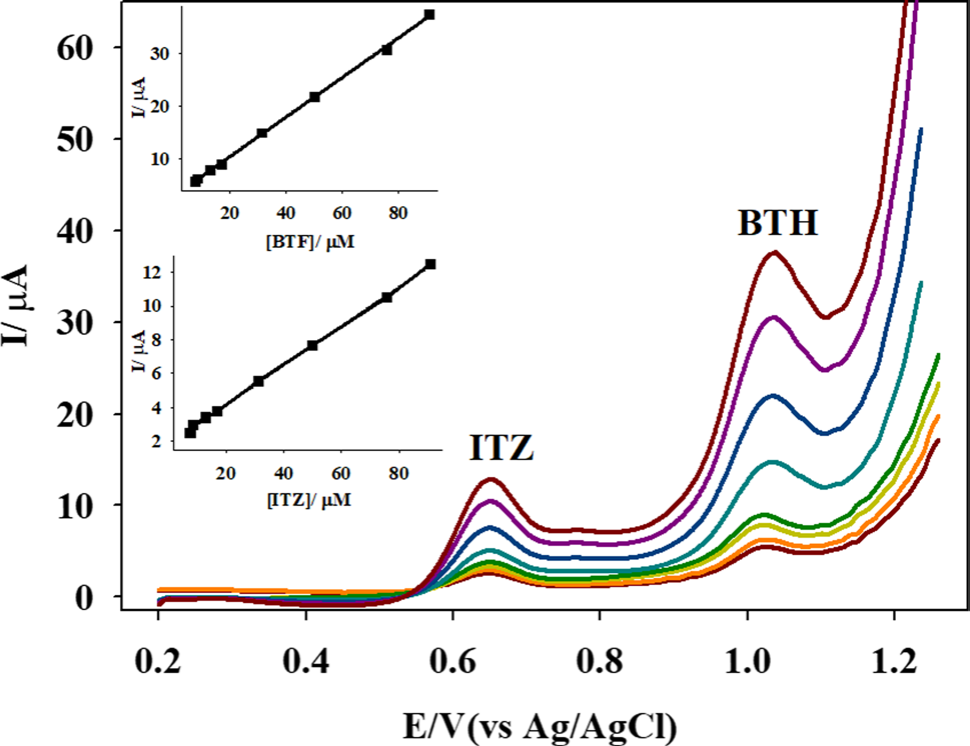
The SWV using Ti-IL/CPE sensor in pH 2.0 B–R buffer corresponding to: 7.65 × 10^−6^–9.091 × 10^−5^ M of both drugs (*v* = 100 mV s^−1^), *P*_H_ = 0.25 V, *P*_W_ = 0.50 V, *S*_H_ = 0.02 V. The inset is the current vs. concentration plot

Ti-IL/CPE was used to record the SWV, while the immediate levels of BTH and ITZ were changed. In the SWVs, anodic peaks with well-defined potentials of 1035.69 and 651.27 mV were observed, which corresponded to the oxidation of BTH and ITZ, respectively, proving that the simultaneous detection of both medicines is attainable at the Ti-IL/CPE, as revealed in Fig. 6. The calibration graphs were linearly associated to BTH and ITZ through the range 7.65 × 10^−6^–9.091 × 10^−5^ M. We calculated the regression equations and concluded that they were as follows:

$${\text{Ip }}\left( {\mu A} \right) \, = \, 0.375 \, C \, \left( {\mu M} \right) \, + \, 2.817; \, R \, = \, 0.9995{\text{ for BTH}}$$

$${\text{Ip }}\left( {\mu A} \right) \, = \, 0.1165 \, C \, \left( {\mu M} \right) \, + \, 1.815; \, R \, = \, 0.9994{\text{ for ITZ}}$$

The value of BHT sensitivity in case of the single determination (0.3671 μA/µM) is extremely close to that observed in the case of a combination (0.3759 μA/µM) including BTH and ITZ. According to the results, BTH and ITZ oxidation are independent, and concurrent assessment of the combination of BTH and ITZ is accurate and selective.

Comparing to the previous electrochemical potentiometric determination of BTH, the present voltammetric work showed the lowest LOD and the wider dynamic range, Table 3.

**Table 3:** Comparison of different modified electrodes for BTH determination

Electrode	Linear range (M)	Detection limit (M)	Method	References
Tricresylphosphate-CPE	1.57 × 10^–7^–1.0 × 10^–2^	1.0 × 10^–7^	Potentiometry	Ali et al. (2020)
SPE	1.0 × 10^−6^–1.0 × 10^−2^	4.0 × 10^−7^	Potentiometry	Frag et al. (2020)
Ti-IL/CPE	2.21 × 10^–7^–13.46 × 10^–5^	61.63 × 10^−9^	Voltammetry, SWV	The present work

## Conclusion

The measurement of BTH in drug substance, pharmaceutical preparations, and real samples were done using a carbon paste electrode enhanced with Ti nanoparticles and ionic liquid in this work. The voltammetric responses of the CV and SWV tests revealed effective electrochemical activity, high sensitivity, selectivity, and repeatability. The technique was used to measure BTH in drug substance, drug products, and real-life samples. The suggested method's low detection limit, along with the convenience of electrode fabrication, make it ideal for reliable drug determination in regular quality control work, and the findings demonstrate that the proposed sensor is capable of investigating the dermatopharmacokinetics of BTH.

## Supplementary Information

Below is the link to the electronic supplementary material.

Supplementary file1 (DOCX 454 kb)
