# Supplementary material for: Sensitive and effective electrochemical determination of butenafine in the presence of itraconazole using titanium nanoparticles-ionic liquid based nanocomposite sensor
Source: Chem Zvesti. 2022 Dec 5;77(4):1929–39. doi: 10.1007/s11696-022-02593-3 (PMC9734920; doi:10.1007/s11696-022-02593-3)
Supplement: Supplementary file 1 — Supplementary file1 (DOCX 454 kb) [file 11696_2022_2593_MOESM1_ESM.docx]

**Electronic Supplementary Information**

**Sensitive and effective electrochemical determination of butenafine in the presence of itraconazole using** **titanium nanoparticles -ionic liquid based nanocomposite sensor**

Mona A Mohamed^1*^, Nahla N. Salama^1^, Maha A. Sultan ^2^, Hadeer F. Manie^1^, Maha M. Abou El-Alamin ^2^

***^1^****Pharmaceutical Chemistry Department, National Organization for Drug Control and Research, Egyptian Drug Authority (EDA)*

*^2^Department of Pharmaceutical Analytical Chemistry, Faculty of Pharmacy, Helwan University, Cairo, Egypt*


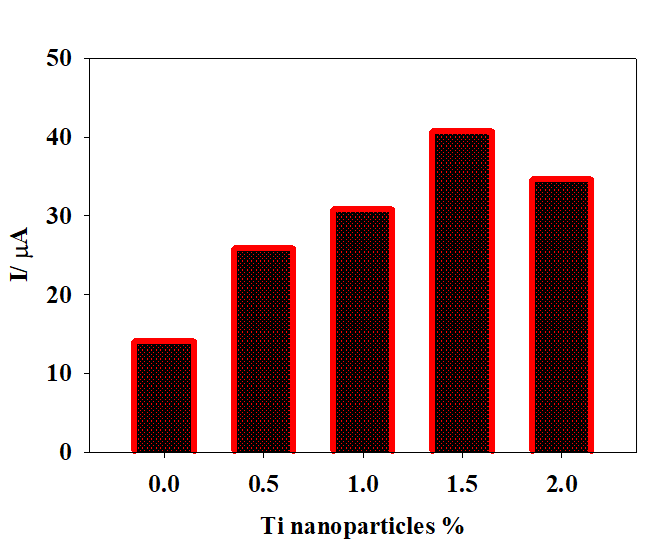


**Fig. S1.** Effect of different Ti nanoparticles percentages used to fabricate CPE.


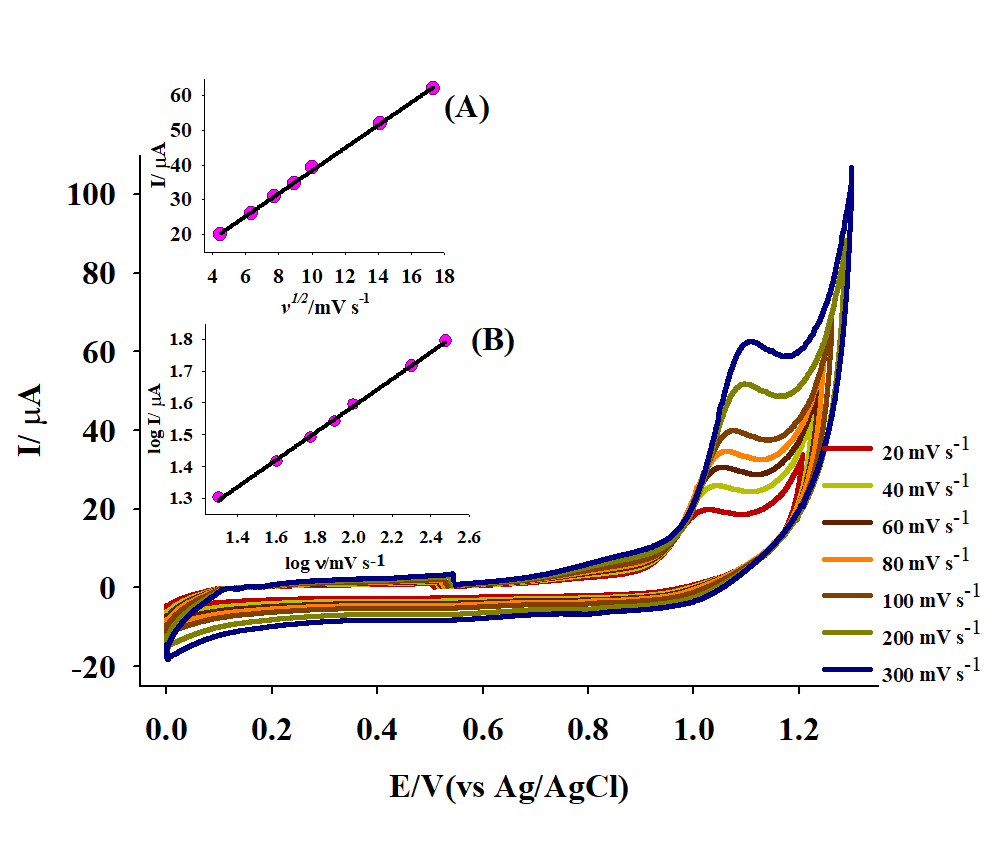


**Fig. S2.** CVs of 0.10 mM of BTH in pH 2.0 B-R buffer (0.04 M) using a Ti-IL/CPE sensor recorded at various scan rates: 20.0-300.0 mVs^-1^. Inset A: the plot of peak current vs. square root of scan rate and Inset B: the plot of log current vs. log scan rate.


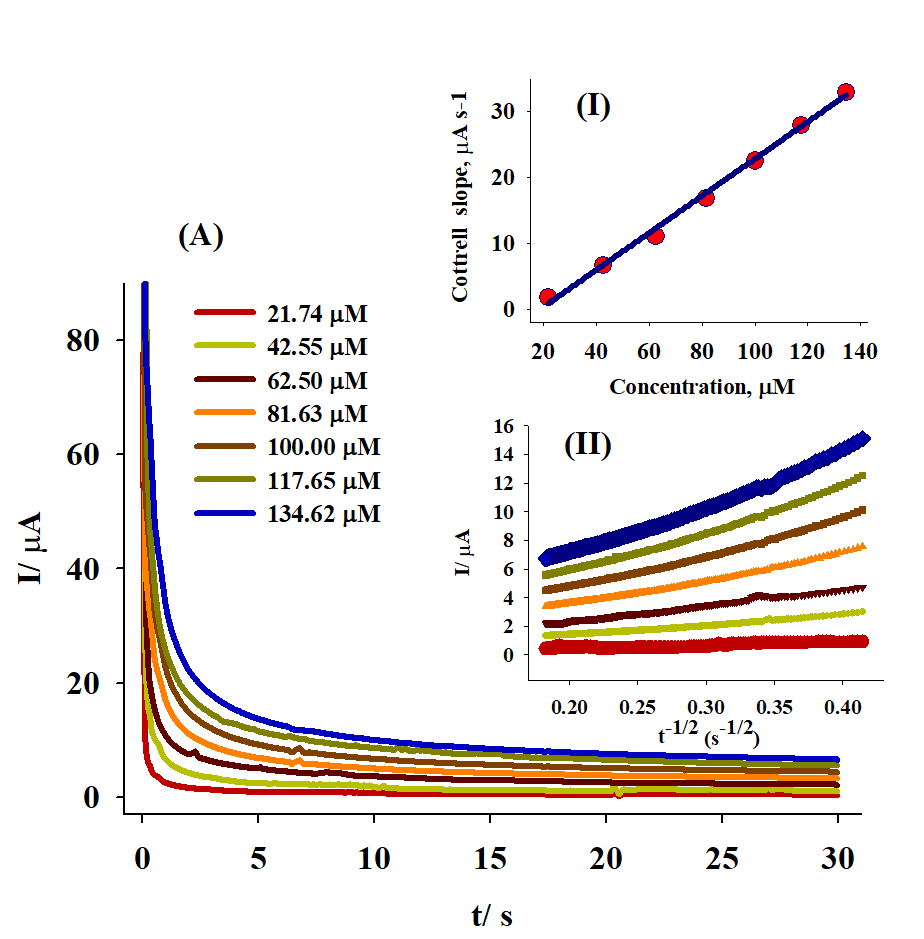

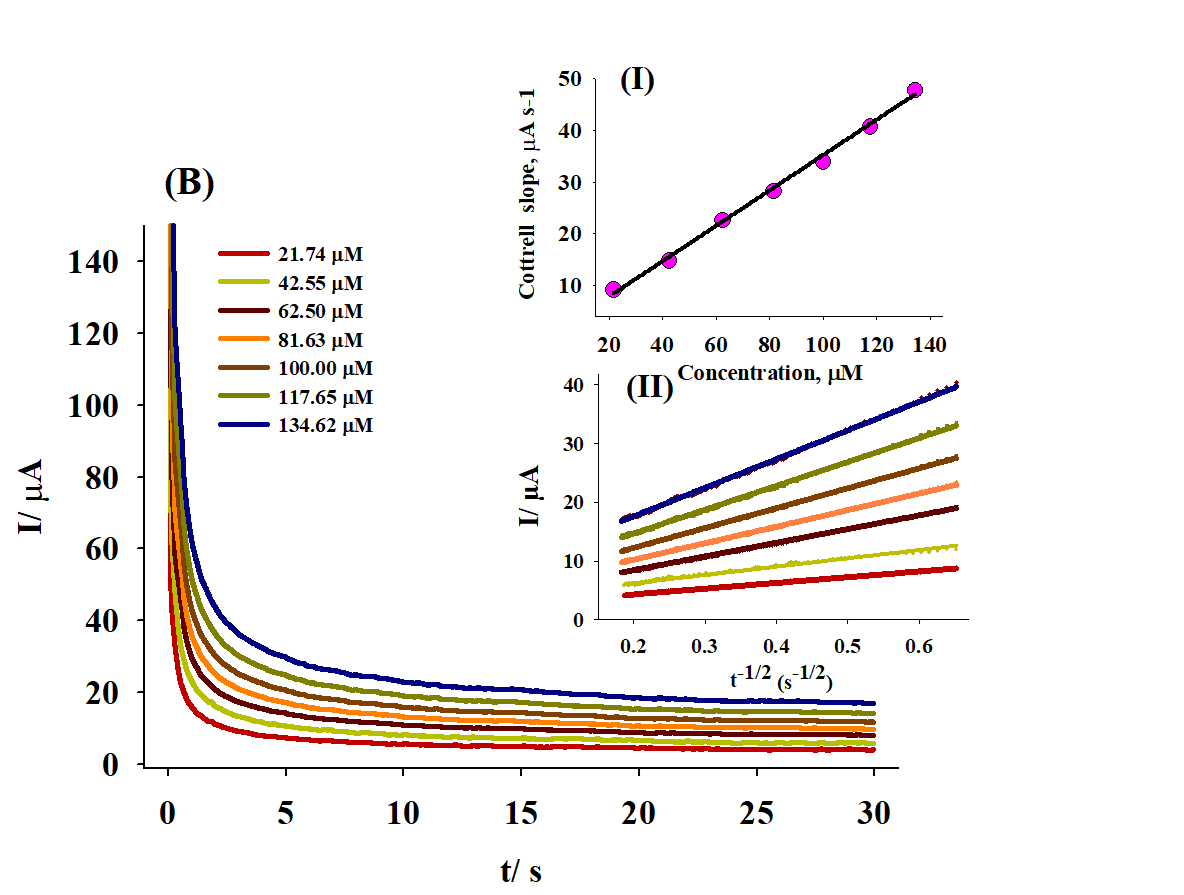


**Fig. S3.** Chronoamperograms for the oxidation of different concentrations of BTH at: (A) Unmodified/CPE and (B) Ti-IL/CPE surfaces in B-R buffer, pH 2.0, for a potential step of 1103.00 mV vs. Ag/AgCl. The numbers 1 to 6 in Cottrell's plot (insets) correspond to 21.74, 42.55, 62.50, 81.63, 100.0, 117.65, and 134.62 µM of BTH, respectively. Insets show the variation of chronoamperometric currents at t=30.00 s vs. BTH concentration.

**
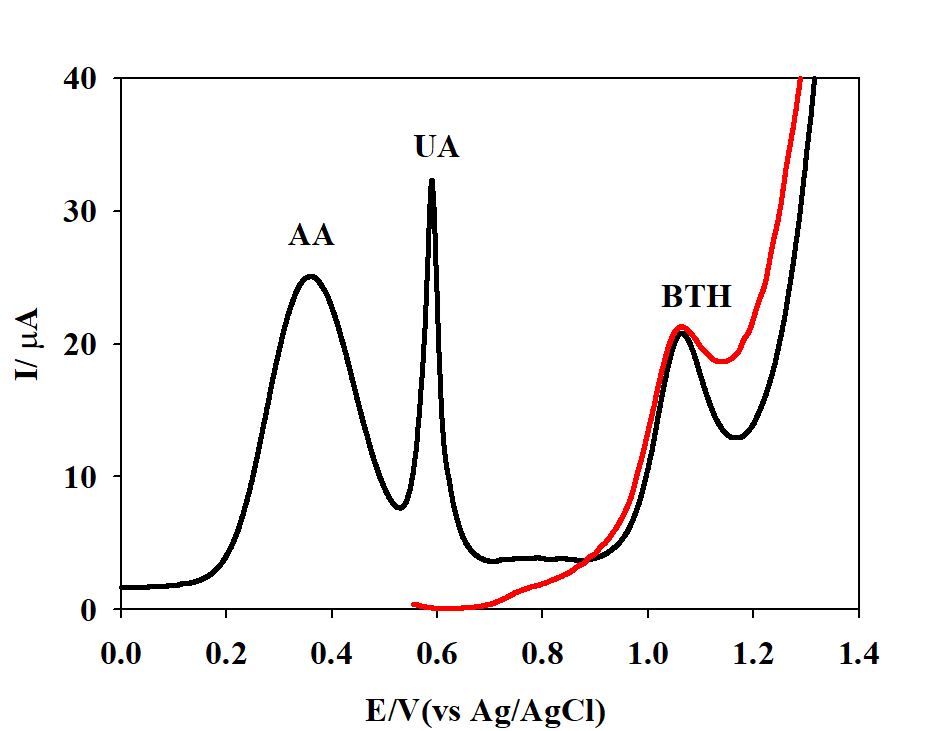
**

**Fig. S4**. SWVs using the Ti-IL/CPE for the sensing of BTH (47.62 µM) in presence of AA (476.0 µM) and UA (476.0 µM) in 0.04 M B-R buffer pH 2.0 at scan rate 100.0 mVs^─1^.

**Table S1.** Analytical parameters and validation results for the determination of BTH in 0.04 M B-R buffer pH 2.0 at Ti-IL/CPE using proposed SWV method (*n* = 3)

| **Analytical parameters** | **Values** |
| --- | --- |
| **Linearity range (M)** | 2.210×10-^7^– 13.46×10^-5^ M |
| **Regression parameters** |  |
| Intercept (µA) | 3.120 |
| Slope (µA µM^-1^) | 0.367 |
| Standard deviation of intercept (µA) | 0.057 |
| Standard deviation of slope (µA µM^-1^) | 0.042 |
| correlation coefficient (R) | 0.9997 |
| **Accuracy** |  |
| (Mean* ± SD) | 99.51 ±0.778 |
| **Precision**** |  |
| Repeatability | 0.761 |
| Intermediate precision | 0.5833 |
| **LOD (M)** | 6.163×10^-8^ |
| **LOQ (M)** | 2.054 ×10^-7^ |

*Mean of five different determinations.

**n=9.

**Table S2.** Statistical comparison of the suggested approach to the production method for determining BTH

| **Parameters** | **Drug substances** | | **Drug product**  **Butaximark 10 mg/1 gm** | |
| --- | --- | --- | --- | --- |
|  | **Proposed**  **method** | **Manufacturer^a^**  **method** | **Proposed**  **method** | **Manufacturer^a^**  **method** |
| **Mean^b^** | 99.52 | 99.29 | 99.15 | 98.95 |
| **SD** | 0.779 | 0.610 | 0.478 | 0.435 |
| **N** | 5 | 5 | 5 | 5 |
| **Variance** | 0.607 | 0.372 | 0.228 | 0.189 |
| **SE** | 0.348 | 0.273 | 0.214 | 0.195 |
| **t-value (2.306)^c^** | 0.519 | ------ | 0.692 | ------ |
| **F-value (6.390 )^c^** | 1.632 | ------ | 1.206 | ------ |

**^a^** Manufacturer HPLC method for drug substance using Inertsil ODS 5 μ, 150 × 4.6 mm as column and methanol, phosphate buffer (4 gm of potassium dihydrogen phosphate in 500 mL water), acetonitrile in ratio;50:450:500 (v/v/v) as mobile phase with flow rate 2 mL/min, injection volume 2.0 μL and detection at 220 nm.
**^b^** Mean of five determinations at four different concentrations.
**^c^** The values between parenthesis are the theoretical values of t and F at (P = 0.05).

**Table S3.** Repeatability and intermediate precision of the proposed SWV method for the determination of BTH in drug substance

|  | **Intra-day (RSD %)** | | **Inter-day (RSD %)** | |
| --- | --- | --- | --- | --- |
| **Taken (µM)** | **Found (µM)±SD** | **Precision (RSD %)** | **Found (µM)±SD** | **Precision (RSD %)** |
| 10.98 | 10.88±0.083 | 0.762 | 11.03**±**0.097 | 0.879 |
| 19.61 | 19.57±0.162 | 0.827 | 19.53±0.064 | 0.327 |
| 32.25 | 32.29±0.226 | 0.699 | 32.24± 0.175 | 0.542 |
